# Selection Signatures in the Genome of Dzhalgin Merino Sheep Breed

**DOI:** 10.3390/ani15192871

**Published:** 2025-09-30

**Authors:** Alexander Krivoruchko, Olesya Yatsyk, Antonina Skokova, Elena Safaryan, Ludmila Usai, Anastasia Kanibolotskaya

**Affiliations:** 1 Federal State Budgetary Scientific Institution “North Caucasian Federal Scientific Agrarian Center”, 356241 Mikhailovsk, Russia; malteze@mail.ru (O.Y.); antoninaskokova@mail.ru (A.S.); telegina.helen@yandex.ru (E.S.); dorohin.2012@inbox.ru (A.K.); 2 Federal State Autonomous Educational Institution for Higher Education, “North Caucasian Federal University”, 355017 Stavropol, Russia; 3 Federal State Autonomous Educational Institution of Higher Education I.M. Sechenov First Moscow State Medical University of the Ministry of Healthcare of the Russian Federation (Sechenov University), 119991 Moscow, Russia; usay_1_i@staff.sechenov.ru

**Keywords:** sheep, Merino, Dzhalgin Merino, genome, SNP, candidate gene, selection signatures

## Abstract

**Simple Summary:**

Selection acting on farm animal populations results in the formation of specific regions in their genomes that reflect the influence of the selection process. Studying such regions allows us to identify genes and genetic variants that play a key role in animal adaptation to environmental conditions and in the formation of productive traits. The Dzhalgin Merino breed was bred in Russia relatively recently and is characterized by high meat and wool productivity, as well as good adaptability to arid steppe conditions. Given the genetic closeness of this breed to the Australian Merino and Rambouillet, the aim of this study was to search for selection signatures and identify candidate genes at loci under selection pressure based on a comparative analysis of the Dzhalgin Merino genomes with Australian Merino and Rambouillet sheep. The identified genes are potentially involved in the formation of adaptive and productive traits significant for this breed. In total, 185 genes were identified in loci showing evidence of selection. Among them, *EPHA6*, *MLLT3*, *ROBO1*, *KIAA0753*, *MED31*, *SLC13A5*, and *ELAVL4* were highlighted as the most promising candidates for further research, due to their association with biological processes such as growth, development, and reproduction.

**Abstract:**

Analysis of selection signatures in the genomes of farm animals enables the detection of genomic regions affected by selection and contributes to the identification of genes underlying adaptive and productive traits. This research aimed to identify loci under selection pressure and to detect candidate genes in Dzhalgin Merino sheep by performing a comparative genomic analysis with the related Australian Merino and Rambouillet breeds. A total of 293 animals were included in the analysis, comprising Dzhalgin Merino (n = 53), Australian Merino (n = 50), Australian Industry Merino (n = 88), and Rambouillet (n = 102). Whole-genome SNP genotyping data for Dzhalgin Merino were generated within this study, while data for Australian Merino, Australian Industry Merino, and Rambouillet were obtained from the SheepHapMap project. For the purposes of analysis, Australian Merino and Australian Industry Merino were combined into a single group (n = 138). To enhance the reliability of the results, three independent methods were employed to detect selection signatures: the fixation index (FST), analysis of linkage disequilibrium variation (varLD), and the cross-population number of segregating sites by length (xp-nSL). The study showed that Dzhalgin Merino have unique genetic signatures potentially associated with adaptation and productivity, which opens up new opportunities for their selection. The identified genes can become the basis for developing new breeding programs aimed at improving both the productive qualities and the adaptive abilities of the breed. Further research should be aimed at a detailed investigation of gene structure within loci under selection pressure and at clarifying the mechanisms by which these genes influence animal phenotypes. A total of 185 genes were identified within genomic regions exhibiting selection signatures. Among these, particular attention was given to *EPHA6*, *MLLT3*, *ROBO1*, *KIAA0753*, *MED31*, *SLC13A5*, and *ELAVL4*, which are involved in biological processes such as growth, development, and reproduction. The identified genes represent potential targets for breeding programs aimed at increasing productivity and adaptive capacity of the breed.

## 1. Introduction

Positive selection acting on farm animal populations leaves characteristic imprints on their genomes, namely at loci under selection pressure. Such imprints, selection signatures, can be used to identify genes and genetic variants that play a key role in animal adaptation to specific environmental conditions or changes in their productive qualities [1]. Currently, many methods have been developed to identify genomic signatures of selection. The most reliable results are provided by an integrated approach in which loci under selection pressure are identified using several independent methods of analysis [2].

According to the literature, when conducting studies aimed at searching for selection signatures in sheep genomes, 2–3 different types of analysis are often combined. Thus, Liu Z. et al. (2022) [3] searched for selection signatures in South African Merino sheep using the FST (the fixation index), iHS (Integrated haplotype score) and xp-EHH (Cross-population extended haplotype homozygosity) methods. Among the most interesting genes located in loci under selection pressure, they identified *GHR*, *LCORL*, *SMO*, *NCAPG*, *DCC*, *IBSP*, *PPARGC1A*, *PACRGL*, *PRDM5*, *XYLB*, *AHCYL2*, *TEFM*, *AFG1L* and *FAM184B*, involved in growth processes that affect carcass characteristics and meat quality [3]. Selection signatures potentially associated with milk, wool and meat productivity, as well as resistance to parasitosis, have been identified in Slovak national breeds using ROH (Runs of homozygosity) and LD (linkage disequilibrium) methods [4]. In a study by Eydivandi S. et al. (2021) [5], simultaneous application of FST and xp-EHH methods in sheep populations from the Middle East and Europe identified genomic regions under selection pressure containing the genes *CIDEA*, *HHATL*, *MGST1*, *FADS1*, *RTL1* and *DGKG*. Both FST and xp-EHH approaches identified 60 common genes as selection signatures, including four candidate genes (*NT5E*, *ADA2*, *C8A* and *C8B*) that were enriched for two significant Gene Ontology (GO) terms related to the adenosine metabolism pathway [5]. Identification of selection signatures in Indian sheep breeds was carried out using ROH and iHS. The results revealed selection signatures in 37 genomic regions mapping to 188 genes, including the cold adaptation gene *TRPM8*, meat quality traits related genes *JADE2*, *PLEKHB2*, *SPP2*, *TSHR* and *UBE2B* and fertility-related gene *PPP3CA* [6]. The use of xp-nSL (cross-population number of segregating sites by length) methods in combination with ROH in Chinese sheep breeds to identify selection signatures allowed the identification of loci associated with domestication and reproduction (*TSHR*, *GTF2A1*, *KITLG*, *FETUB*, *HNRNPA1*, *DCUN1D1*, *HRG*) [7].

Genomic selection signatures in Russian sheep breeds have not been extensively investigated. A study devoted to identifying selection traits associated with acclimatization and economically important traits in 15 Russian sheep breeds was conducted by Yurchenko, A.A and co-authors [8]. The study analyzed the genomes of the Buubey, Lezgin, Karachay, Karakul, Tuvan, Edilbaevskaya, Romanov, Russian Long-Wool, Altai Mountain, Grozny, Salskaya, Volgograd, Krasnoyarsk, Baikal, and Kulunda sheep breeds. The study identified genomic regions that are presumably under selection pressure. These regions contain known candidate genes associated with morphology, adaptation, and domestication (*KITLG*, *KIT*, *MITF*, and *MC1R*), wool productivity (*DSG2*, *DSC2*, and *KRT2*), reproduction (*CMTM6*, *HTRA1*, *GNAQ*, *UBQLN1*, and *IFT88*), milk production (*ABCG2*, *SPP1*, ACSS1, and *ACSS2*), etc. [8].

Studying the sheep genome to detect selection signatures is a promising area of scientific research, since the results obtained not only help to understand the genetic basis of adaptation, but also provide an idea of the genetic architecture of phenotypic traits and can be used to improve breeding programs. Historically, the bulk of the sheep population in Russia is represented by fine-fleeced breeds. In this regard, the search for selection signatures in merino sheep is of particular interest. Of great interest is the search for selection signatures in breeds bred for use in special climatic and natural conditions. In this case, it is possible to identify genomic loci associated with adaptation to temperature, nutrition and maintenance. One of such breeds is the Dzhalgin Merino. The breed was bred in the Stavropol Territory of the Russian Federation in 2013. It is distinguished by high rates of meat and wool productivity, good adaptability to the conditions of dry steppes. The main method of creating the breed is reproductive crossing. The mother stock for breeding the Dzhalgin Merino sheep was the Stavropol breed, and the paternal form was the Australian Merino and Caucasian breeds. Importantly, the Stavropol breed itself has a composite origin, as it was developed by crossing Mazaev and Novokavkaz ewes with American Rambouillet and Australian Merino rams, with the simultaneous use of Novokavkaz and Mazaev rams [9]. Thus, Dzhalgin Merino is genetically related to Australian Merino both directly through paternal ancestry and through the Stavropol breed, and to Rambouillet through the Stavropol breed. Given the genetic relatedness of Dzhalgin Merino sheep to both Australian Merino and Rambouillet breeds, investigating their genetic differentiation and identifying interpopulation selection signatures is of particular interest.

The aim of our study was to search for selection signatures and identify candidate genes in loci under selection pressure, potentially playing an important role in the adaptive and productive qualities of Dzhalgin Merino sheep.

## 2. Materials and Methods

### 2.1. Ethics Statement

The sample collection and study purpose were approved by the Institutional Animal Care and Use Committee (approval number 2021–0047, 10 November 2021) of the All-Russian Research Institute of Sheep and Goat Breeding, Stavropol, Russian Federation.

### 2.2. Experimental Animals and Sample Collection

A total of 293 animals were included in the analysis as the object of the study, all of which underwent whole-genome SNP genotyping. The sample comprised Dzhalgin Merino (DZM, n = 53), Australian Merino (AM, n = 138), and Rambouillet (Ramb, n = 102).

Whole-genome SNP genotyping data for Dzhalgin Merino were generated within the framework of this study, while the data for Australian Merino and Rambouillet were obtained from the SheepHapMap project, where animals had been genotyped using the Illumina OvineSNP50 BeadChip (Illumina, San Diego, CA, USA). The group of Australian Merino included Australian Merino (n = 50) and Australian Industry Merino (n = 88). The genotyping data of the SheepHapMap project [10] were obtained from the Web-Interfaced next-generation Database dedicated to genetic Diversity Exploration (http://widde.toulouse.inra.fr, accessed on 28 September 2025).

### 2.3. Genotyping and Genotyping Quality Control

Genotyping of Dzhalgin Merino animals was performed using Ovine Infinium HD BeadChip 600 K (Illumina, San Diego, CA, USA) according to the manufacturer’s protocol. Primary processing of genotyping results was performed using Genome Studio 2.0 software (Illumina, San Diego, CA, USA).

The quality of our own genotyping data was controlled using PLINK V.1.09 software [9]. Samples in which the number of lost genotypes did not exceed 10% were included in the processing. For further analysis, we used only autosomal biallelic SNPs. SNPs without chromosomal or physical localization, SNPs with a frequency of lost genotypes exceeding 10%, and those that exceeded the threshold of deviation from Hardy–Weinberg equilibrium (HWE) *p* = 0.000001 were excluded. Since genotyping of the analyzed sheep breeds was performed using chips of different densities (50 k and 600 k), only polymorphisms present on both chip types were used when forming the combined dataset. Substitution positions were updated in accordance with the ARS-UI_Ramb_v2.0 genome assembly using the PLINK v1.09 program [11]. The combined dataset was subjected to quality control according to the same criteria described above. The final dataset contained data on 293 samples and 38,804 SNPs.

### 2.4. Principal Component Analysis

For the principal component analysis we used 36,615 SNPs, after linkage disequilibrium pruning (window size 50 SNPs, step 5 SNPs, threshold r^2^ > 0.5), performed using the PLINK V.1.09 software [11]. Principal component analysis was performed in the R v. 4.3.2 environment using the SNPRelate package [12].

### 2.5. Search for Selection Signatures

Calculation of the Weir and Cockerham weighted FST values for pairwise comparison of the three analyzed groups was performed using VCFtools v0.1.16 software [13], with a set window size of 100 kb and step size of 10 kb. Signals included in the upper 1% of the weighted FST values were considered as signs of selection between the two breeds.

VarLD v.1.0 software was used to assess differences in linkage disequilibrium patterns between the analyzed groups [14]. LD values calculated for sliding windows containing 50 SNPs with minor allele frequency above 5% within each autosome were transformed into standardized varLD scores, from which signatures of selection were identified. Calculations of standardized values for varLD scores, as well as visualization, were performed in R using scripts provided by the varLD authors. Signals in the top 1% of standardized scores were considered as signatures of selection between the two breeds.

xp-nSL statistics were calculated using Selscan v.2.0 with default parameters [15]. The required haplotypes were obtained by phasing using the SHAPEIT v.4.1.3 software [16]. The xp-nSL analysis results were normalized using the norm v 1.3.0 software. Positive XP-nSL values indicated the presence of hard or soft selection in the Dzhalgin Merino population, while negative values indicated the presence of hard or soft selection in the comparison populations. Since we were primarily interested in the selection features in the Dzhalgin Merino group, the signals included in the upper 1% of positive normalized xp-nSL estimates were considered as selection features. Annotation of genes located in loci under selection pressure was performed in windows of +/− 150,000 bp from the SNPs that showed selection features.

### 2.6. Gene Annotation and Construction of Gene Networks

Gene annotation was performed using the biomaRt package in the R environment [17].

Enrichment analysis was performed using the ShinyGO 0.80 platform [18]. The significance threshold for the false discovery rate (FDR) = 0.05. Gene networks were constructed using the platform https://string-db.org, accessed on 28 September 2025.

### 2.7. Visualization

Graphs were plotted in the R environment using the qqman, ggplot2, and VennDiagram packages.

## 3. Results

### 3.1. Genetic Differentiation of the Studied Groups Using PCA and FST Methods

**Figure 1:**
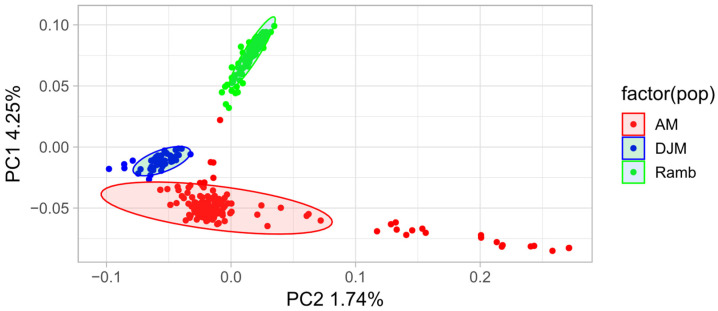
Principal components analysis (PCA) showing the genetic differentiation among Dzhalgin Merino (DZM), Australian Merino (AM), and Rambouillet (Ramb) sheep groups.

According to the PCA results, Rambouillet and Dzhalgin Merino are presented as well-consolidated clusters, while AM showed greater intrabreed genetic variability compared to DZM and Ramb (Figure 1). In the PCA analysis, PC1 (the first principal component) accounted for 4.25% of the total genetic variability, separating all three analyzed groups of sheep, while PC2 (the second principal component) represented only 1.74% of the total genetic variability.

In the course of the research, the Weir and Cockerham FST fixation indices were calculated. Table 1 shows the weighted values for the analyzed populations. As expected, a low degree of differentiation was noted between all analyzed groups. Animals from the Australian Merino group were the closest to the Dzhalgin Merino. The greatest genetic distance was observed between the Rambouillet and the Australian Merino group. This is consistent with the results of the principal component analysis.

### 3.2. Search for Selection Signatures

**Figure 2:**
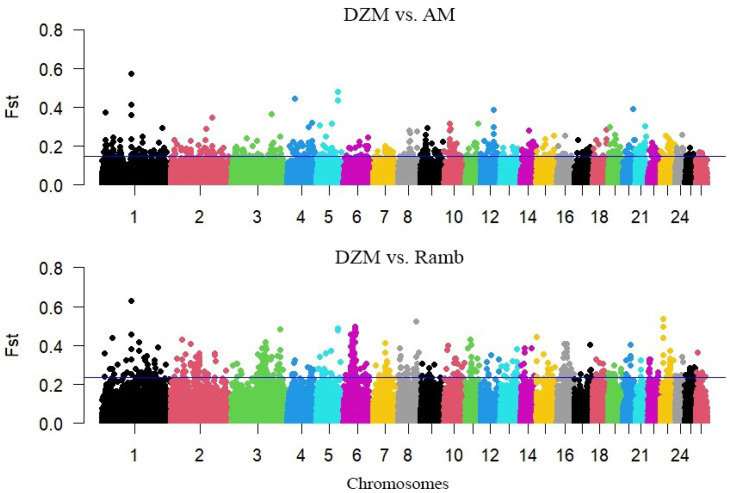
Manhattan plot of fixation index values used to identify genomic regions under selection pressure in comparisons between Dzhalginsky Merino and Australian Merino (DZM vs. AM), and between Dzhalginsky Merino and Rambouillet (DZM vs. Ramb). The horizontal blue line marks the 99th percentile of the FST values, representing the threshold for regions considered to be under selection pressure.

Based on the calculations of the weighted FST values between the Dzhalgin Merino and Australian Merino groups, 2089 windows were identified that exceeded the 99% percentile with a value of 0.147479. The detected windows contained 451 protein-coding genes. When determining the selection features by FST between the Dzhalgin Merino and Rambouillet, the 99% percentile was 0.235915. This threshold was exceeded by 2087 windows, in which 454 protein-coding genes were identified. In both cases, the highest weighted FST value was identified for the window 1:127,080,001–127,180,000 on chromosome 1 (Figure 2). This window overlaps part of the *GRIK1* gene.

**Figure 3:**
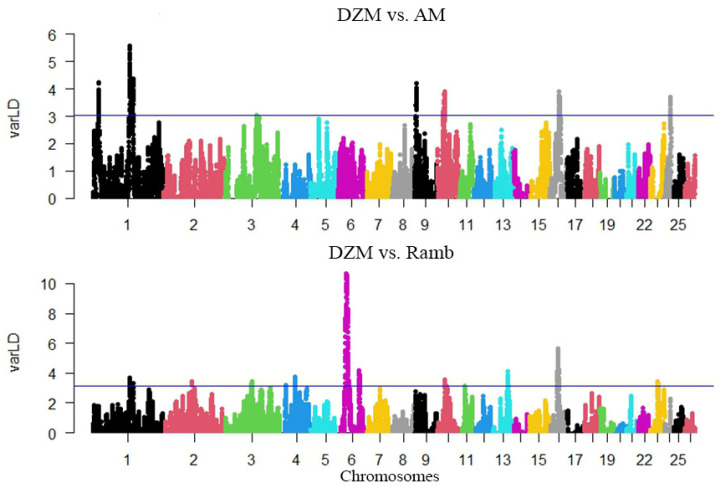
Manhattan plot of VarLD values illustrating genomic regions with differences in linkage disequilibrium patterns between Dzhalginsky Merino and Australian Merino (DZM vs. AM), and between Dzhalginsky Merino and Rambouillet (DZM vs. Ramb). The horizontal blue line marks the 99th percentile of the VarLD values, representing the threshold for regions considered to be under selection pressure.

Based on the calculations of the linkage disequilibrium values between the Dzhalgin and Australian Merino groups, 347 windows were identified that were included in 1% of the maximum standardized LD values. A total of 289 genes were identified in these windows. The highest LD value, which was 4.721373, was determined for the window located on chromosome 1; the coordinates of the window center were 1:147,516,958 (Figure 3). This point falls within the area of the *ROBO1* gene. Based on the calculations of the linkage disequilibrium values between the Dzhalgin Merino and Rambouillet groups, 338 windows were identified that were included in 1% of the maximum standardized LD values. A total of 483 genes were identified in these windows. The maximum standardized LD value was 11.94841853 and was found for the window located on chromosome 6; the coordinates of the middle of the window are 6:37,577,437. This point is located in the intergenic space (Figure 3).

**Figure 4:**
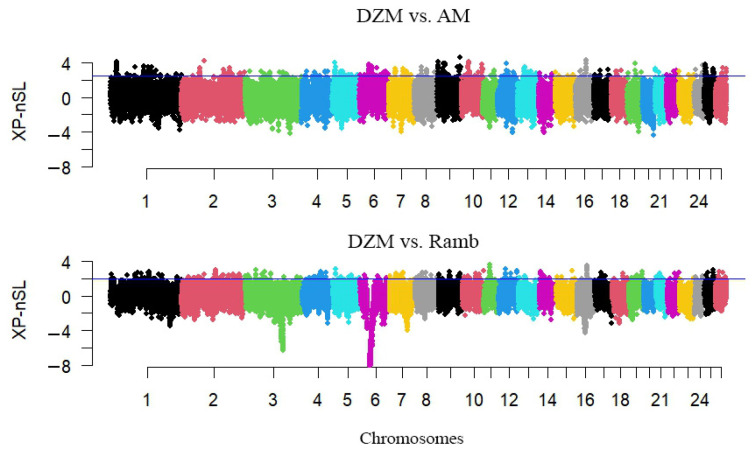
Manhattan plot of xp-nSL scores used to detect selection signatures in the Dzhalgin Merino in comparison with Australian Merino (DZM vs. AM) and Rambouillet (DZM vs. Ramb) sheep groups. The horizontal blue line marks the 99th percentile of the xp-nSL values, representing the threshold for regions considered to be under selection pressure.

According to the calculation results of xp-nSL statistics between the Dzhalgin Merino and Australian Merino groups, the highest normalized xp-nSL value of 4.61038 was observed for the OAR9_95091169.1 substitution located on chromosome 9 (Figure 4). The detected polymorphism is located in the intergenic region. The established threshold corresponding to the 99th percentile was exceeded by 364 SNPs. 468 genes were identified in the adjacent regions.

For the Dzhalgin Merino and Rambouillet groups, the maximum normalized xp-nSL value was 3.62782 for the s45020.1 substitution located on chromosome 11 (Figure 4). This substitution is located in the intergenic region. The established threshold of the 99th percentile was exceeded by 360 SNPs. 484 genes were found in the adjacent regions.

**Figure 5:**
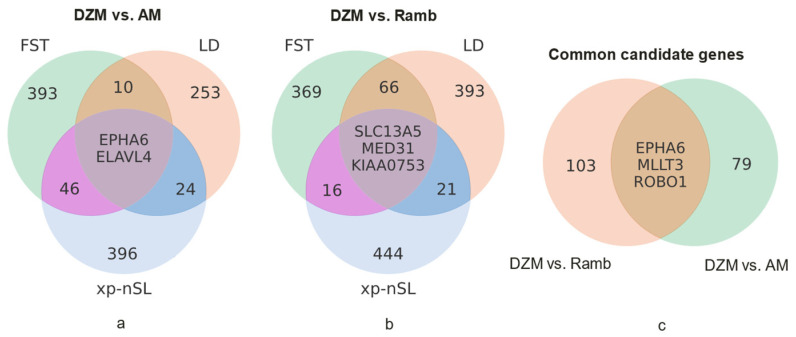
Overlapping genes at loci under selection pressure identified by different methods in comparative analysis of the genomes of Dzhalgin Merino and Australian Merino (**a**), Dzhalgin Merino and Rambouillet (**b**), as well as genes selected for further analysis and common to the candidate gene networks “DZM vs. AM” and “DZM vs. Ramb” (**c**).

To increase the reliability of identifying candidate genes in loci under selection pressure, we selected for further analysis only those genes that were located in genomic regions with selection signatures confirmed by at least two methods in each comparison (Figure 5). The selected genes were combined into gene networks. As a result, when comparing Dzhalgin Merino with Australian ones (the “DZM vs. AM” network), 82 candidate genes were identified (Supplementary Table S1, Supplementary Figure S1). In this case, the selection signatures in loci including the *EPHA6* and *ELAVL4* genes were confirmed by three methods at once. When comparing the Dzhalgin Merino with the Rambouillet (the DZM vs. Ramb network), 106 candidate genes were identified (Supplementary Table S2, Supplementary Figure S2), and in three of them (*SLC13A5*, *MED31*, *KIAA0753*) the presence of selection was confirmed by all three methods. Both formed networks have three common genes: *EPHA6*, *MLLT3* and *ROBO1*.

### 3.3. Evaluation of PPI Interactions and Functional Enrichment of GO Terms

When constructing candidate gene networks that are supposedly under selection pressure using the STRING platform, due to the fact that the functions of most genes and proteins in the sheep organism are less well understood than in the human organism, gene networks were formed for both species at once. In all constructed gene networks, with a minimum required interaction score of medium confidence (0.400), significantly more interactions were found between proteins than would be expected for a random set of proteins of the same size and distribution (Table 2). Such enrichment indicates that the proteins are at least partially biologically related as a group.

To clarify the functions of the identified genes, functional enrichment analysis of gene ontology (GO) categories was performed using the ShinyGO 0.80 platform for molecular functions, biological processes, and cellular components. Since the functions and interactions of the analyzed genes in sheep are poorly understood, functional enrichment analysis was performed using human orthologs.

**Figure 6:**
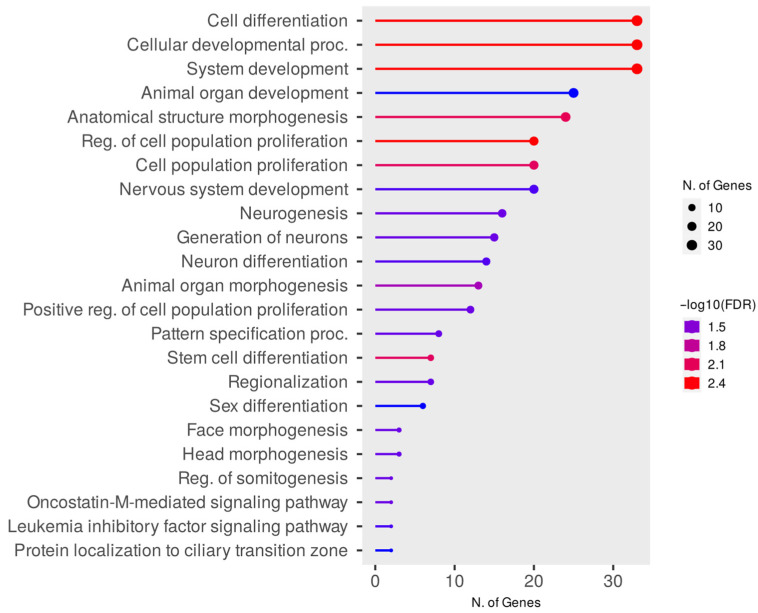
Enrichment of Gene Ontology of Biological Processes for the DZM vs. AM Network.

Significant enrichments for the DZM vs. AM network were identified for 23 terms of gene ontologies of biological processes (Figure 6, Supplementary Table S3) and 4 terms of molecular functions (Supplementary Table S4). No significant enrichments were identified for gene ontologies of cellular components. 50 genes from the DZM vs. AM network are involved in the enriched pathways of biological processes. The largest number of genes in the network are involved in the GO:0030154 cell differentiation, GO:0048731 system development, and GO:0048869 cellular developmental process pathways (FDR= 0.0038), including the *EPHA6* and *ELAVL4* genes. These two genes are involved in eight gene ontologies at once: GO:0048699 generation of neurons, GO:0030182 neuron differentiation, GO:0008283 cell population proliferation, GO:0022008 neurogenesis, GO:0009653 anatomical structure morphogenesis, GO:0007399 nervous system development, GO:0030154 cell differentiation, and GO:0048869 cellular developmental proc. The highest number of enriched terms included genes *DMRTA2* (15), *ROBO1*, and *MSX1* (14).

**Figure 7:**
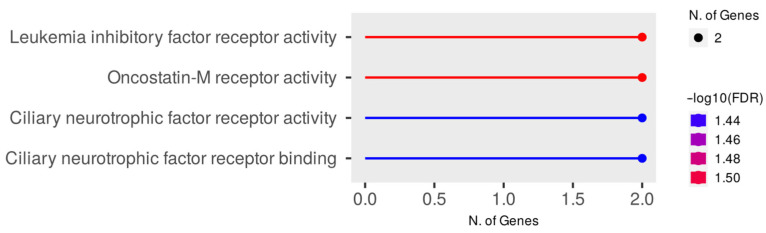
Gene ontology enrichment of molecular functions for the DZM vs. AM network.

When analyzing the enrichment of the DZM vs. AM network according to the gene ontology of molecular functions, it was found that the *LIFR* and *OSMR* genes are involved in the pathways GO:0004923 leukemia inhibitory factor receptor activity, GO:0004924 oncostatin-M receptor activity, GO:0004897 ciliary neurotrophic factor receptor activity, GO:0005127 ciliary neurotrophic factor receptor binding (FDR = 0.0306–0.0365), (Figure 7).

**Figure 8:**
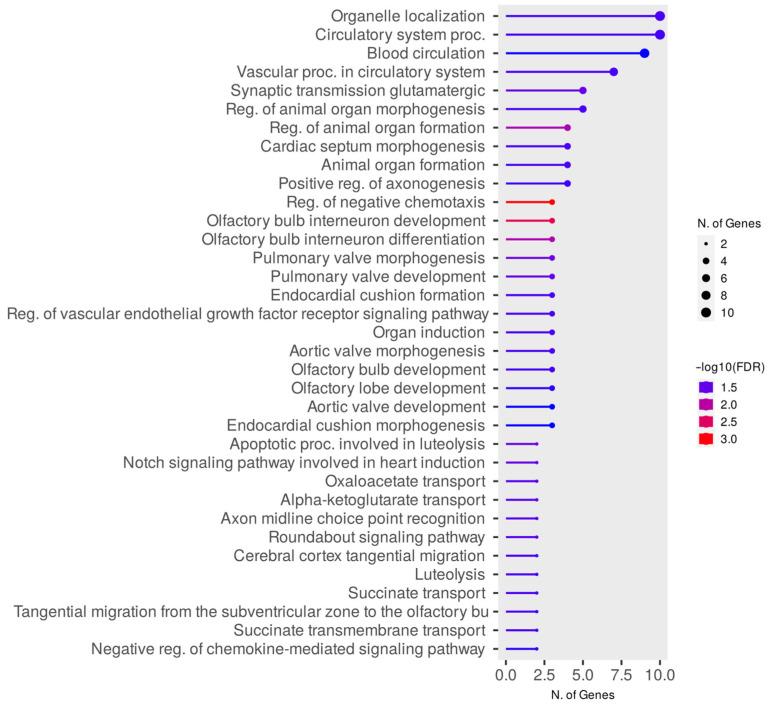
Gene ontology enrichment of biological processes for the DZM vs. Ramb network.

Significant enrichments for the DZM vs. Ramb network were identified for 38 Gene Ontology terms of biological functions (Figure 8, Supplementary Table S5) and 1 term of cellular components (Supplementary Table S6). A total of 29 genes from the DZM vs. Ramb group are involved in the enriched pathways of biological processes. The *ROBO1* gene is involved in 26 terms, *ROBO2* is involved in 24, and *SLIT2* in 20. The largest number of genes are involved in the GO:0051640 organelle localization and GO:0003013 circulatory system process pathways. The lowest FDR value (0.0009) was determined for the GO:0050923 reg. of the negative chemotaxis pathway.

According to the gene ontology of cellular components, significant enrichment was found only for the GO:0005944 phosphatidylinositol 3-kinase complex class IB pathway (FDR = 0.0411). This pathway involves the *PIK3R5* and *PIK3R6* genes.

## 4. Discussion

Knowledge of genetic diversity and genome regions subject to positive selection is one of the promising keys for effective management of genetic resources in sheep breeding. It allows identifying selection signatures associated with important economic traits. In this study, 185 genes located in loci under selection pressure and potentially affecting their adaptive and productive qualities were identified in Dzhalgin Merino sheep using three crosspopulation types of analysis. Comparative analysis of the genomes of Dzhalgin Merino with the genomes of animals of the Australian Merino group revealed 82 genes in loci under selection pressure (DZM vs. AM network), and 106 genes were identified when compared with animals of the Rambouillet group (DZM vs. Ramb network). The *EPHA6*, *MLLT3* and *ROBO1* genes were present in the results of both comparison groups, which emphasizes their breeding importance for the Dzhalgin Merino breed and makes them the most interesting for further study.

The *EPHA6* gene belongs to the ephrin receptor tyrosine kinase family, which plays a key role in intercellular communication and regulation of cell migration, neurogenesis and angiogenesis [19]. As documented in previous studies, the *EPHA6* gene is associated with fertility and reproductive traits in such species of farm animals as goats [20], sheep [21] and cattle [22]. In particular, in Jining grey goats, *EPHA6* was identified as a differentially methylated and differentially expressed gene associated with litter size [20]. GWAS results identify the EPHA6 gene as a candidate gene associated with fertility traits in beef cattle [22] and Polish mountain sheep [21]. The *EPHA6* gene has also been identified as a candidate gene associated with nervous system reactivity and temperament in cattle [23]. In addition, *EPHA6* was identified as a positive selection gene in studies using ROH and his methods in Guishan goats [24]. *EPHA6* is also associated with metabolic processes affecting fatty acid content and meat quality in Nelore cattle [25]. *EPHA6* has also been identified using various genomic approaches as being under intense positive selection for milk production traits, particularly in Holstein cows [26]. Link between *EPHA6* gene and breast meat color in chickens revealed [27]. Thus, despite the lack of precise data on the mechanisms of *EPHA6* influence on the phenotype, it can be assumed that its participation in angiogenesis and neurohumoral regulation can affect the reproductive function and economically important traits of cattle, including traits of milk and meat productivity.

The *ROBO1* gene is part of the SLIT-ROBO signaling pathway, which is involved in the formation and maturation of primordial follicles in the fetal ovaries, which affects the future reproductive potential of the animal. Studies have shown an association of *ROBO1* with antral follicle count in Nelore and Angus heifers, making it an important candidate for fertility assessment [28]. The *ROBO1* gene has also been identified as being under positive selection for wool fineness in sheep [29]. This suggests that *ROBO1* contributes not only to fertility but also to other economically important traits such as wool quality.

The *MLLT3* gene plays a key role in the activation of gene transcription and chromatin remodeling during embryogenesis [30]. In addition, *MLLT3* modifies histone H3K79, which plays an important role in the development of the cerebral cortex [31]. A study on Chongming goats showed differential expression of *MLLT3* between high and low fertility groups [32]. Expression of this gene is also differentially expressed in the bovine endometrium during the luteal and implantation stages, as well as before and after puberty in Bos indicus heifers [33]. The regulatory function of *MLLT3* in chromatin remodeling and transcriptional elongation may provide fine-tuning of the expression of fertility-related genes, making it important for reproductive success and influencing various economically important aspects.

According to the results of our enrichment analysis, the *EPHA6*, *ROBO1*, and *MLLT3* genes are involved in the following biological process library terms: GO:0007166 cell surface receptor signaling pathway, GO:0009653 anatomical structure morphogenesis, GO:0031325 positive regulation of cellular metabolic process, GO:0006464 cellular protein modification process, GO:0036211 protein modification process, GO:0009893 positive regulation of metabolic process, GO:0043412 macromolecule modification, GO:0030154 cell differentiation, GO:0048869 cellular develop-mental process, GO:0048731 system development. The obtained results, together with the data from literary sources, indicate that the *EPHA6*, *ROBO1* and *MLLT3* genes are important for the reproductive qualities of animals and can also affect other economically valuable traits. Their study in the context of genomic selection opens up new prospects for improving the fertility and productivity of farm animals.

Of particular interest is the *ELAVL4* gene, which, along with *EPHA6,* was identified in loci under selection pressure based on the results of a comparative analysis of the genomes of Dzhalgin Merino and Australian Merino using three methods at once: FST, varLD and xp-nSL. The *ELAVL4* gene encodes a protein involved in the regulation of mRNA stability and translation. The main function of *ELAVL4* is to bind to mRNA in neurons and participate in post-transcriptional regulation of genes important for neuronal differentiation, development and maintenance of nervous system functions. The HuD protein, a product of the *ELAVL4* gene, is a key element in the stabilization and transport of mRNA associated with neuronal development, synaptic plasticity and nervous activity [34,35]. In humans, GWAS results identify polymorphisms in the *ELAVL4* gene region associated with body mass index [36]. Direct links between *ELAVL4* and economically important traits in animals have not been identified at present. However, based on its role in regulating the nervous system, it is possible to assume an indirect effect on behavior and learning ability, which may be important for species with pronounced sociality or complex behavioral patterns. Also, its involvement in the formation of neuroplasticity, cognitive functions, and stress response may indirectly affect the adaptive traits of animals.

When comparing the genome of Dzhalgin Merino with the genome of Rambouillet sheep, three genes—*SLC13A5*, *MED31* and *KIAA0753*—were identified at loci under selection pressure, based on the results of all three types of analysis used.

The *SLC13A5* gene encodes a transport protein, the sodium-citrate cotransporter, involved in the transfer of citrate across cell membranes. Citrate plays an important role in metabolism, being a key component for the synthesis of fatty acids and maintaining energy balance, as it is involved in the tricarboxylic acid cycle (Krebs cycle) [37]. Due to the importance of the SLC13A5 gene for energy and fat metabolism, it may also be significant in the context of agricultural animal productivity, where the functioning of metabolic pathways is reflected in live weight and meat quality. The *SLC13A5* gene has previously been proposed as a possible marker of intramuscular fat content in pigs [38].

The *MED31* gene encodes a protein that is part of the Mediator complex, which is a multifunctional complex involved in the regulation of transcription in eukaryotes, as well as regulating key processes of cell proliferation, differentiation and organogenesis. *MED31* is necessary for the correct differentiation of stem cells. When it is knocked out, there is a disruption in the differentiation of cells into certain cell lines, as has been shown in the example of adipogenesis [39]. Studies have shown that *MED31* plays a key role in cell proliferation, especially at the stage of embryonic development. Thus, *MED31* is involved in the regulation of the expression of genes responsible for limb development, such as Sox9 and Col2a1. These genes are necessary for chondrocyte differentiation and bone formation. Mutations in *MED31* lead to disruption of the expression of these genes, which causes delays in limb rudiment development and ossification defects [40]. There is no data on the relationship between the *MED31* gene and productive traits of animals; however, it can be assumed that it may be associated with the amount of fat in the carcass due to its role in the regulation of adipocyte differentiation.

The *KIAA0753* gene encodes a protein involved in cell division, centriole formation, and cilia formation, which play an important role in cell polarity, signaling, and tissue differentiation. This protein is critical for the proper formation of skeletal structures, and mutations in it can lead to ciliopathies—diseases associated with disruption of cilia, which leads to multiple pathologies, including skeletal dysplasia and brain development disorders. There are no data on the association of *KIAA0753* with productivity traits in animals [41,42].

It should be noted that the relatively small sample size used in this study represents a limitation that may increase the risk of bias and limit the statistical power of the analyses; therefore, the results should be interpreted with caution, although comparable sample sizes have been successfully applied in similar genomic studies of sheep and other farm animal species, particularly in research concerning local or small-sized breeds. For example, Adeniyi O.O. et al. (2022) [43], when identifying selection signatures in the Balusha Sheep Breed using the xp-EHH method, used small sample sizes: Balusha (n = 30), Bardhoka (n = 26), Istrian (n = 21), and Ruda (n = 15). As a result, genes involved in melanogenesis and T-cell receptor signaling pathways were identified, which the authors associated with selection for a specific coat color pattern and resistance to certain infectious diseases [43]. Chen S. et al. (2022) [44] detected selection signals by comparing domestic piglets of the Anqing Six-End-White breed (n = 24) with Asian Wild Boar (n = 6). Despite the small sample size, the authors were able to detect reliable selective signals, including the *MSTN, SMPD4*, and *BCL6* genes associated with meat productivity, development, and immune response [44]. In the study by Waineina R.W. et al. (2022) [45], four goat breeds were analyzed: Galla (n = 12), Alpine (n = 29), Saanen (n = 24), and Toggenburg (n = 31). The analysis included iHS, XP-EHH, FLK, and hapFLK, which allowed the identification of loci under selection pressure containing candidate genes related to adaptation, immunity, milk production, and reproductive traits (*HYAL1*, *HYAL3*, *LEPR*, *PDE4B*, *MST1*, *PCK*) [45]. The combined use of various analytical methods to identify selection signatures with a small sample size was also applied by Dzomba et al. (2023) [46]. Their study included sheep breeds with sample sizes ranging from 8 to 52 individuals. The methods used included iHS (within-breed), XP-EHH and Rsb (between-breed), hapFLK, and FST. Despite sample sizes of less than 55 individuals, functions related to immunity, wool color, growth, and adaptation were successfully associated with the detected selection signatures [46].

A specific feature of studies on local breeds is that the available populations for analysis are often small, and forming representative samples is challenging. Nevertheless, such populations are of particular value, as their genetic structure bears traces of long-term adaptation to specific environmental conditions and the results of targeted selection. In our study, the potential limitations associated with the sample size were minimized through the application of an integrated approach combining several independent methods (FST, varLD, xp-nSL) based on different statistical principles. However, FST is a more conservative test that primarily reflects completed or ancient selection processes, accompanied by allele fixation and pronounced inter-population differentiation [47]. varLD identifies changes in local linkage disequilibrium patterns arising during population divergence and breed formation, thereby capturing selection signatures that generally reflect more recent processes than those revealed by FST [48]. In contrast, xp-nSL exhibits high power to capture recent and continuing selection processes, maintaining robustness across a wide spectrum of allele frequencies, including both low and high values [15]. These differences likely explain why the number of candidate genes identified varied among the methods. The limited overlap of the results is therefore an expected consequence of the different sensitivities of the methods and reflects distinct temporal scales of selection, whereas their combined use provides a more comprehensive and reliable identification of genomic regions under selection pressure. The integrative approach applied in this study, combining different statistical methods, enabled us to concentrate on selection signatures validated by multiple criteria. Therefore, while the limited sample size requires cautious interpretation of the findings, the applied integrated analytical approach enhances their robustness and provides valuable insights into selection signatures in the Dzhalgin Merino sheep breed.

## 5. Conclusions

The obtained results indicate the presence of characteristic selection signatures in the genome of the Dzhalgin Merino breed, reflecting the influence of targeted selection on the development of adaptive and productive traits. These genetic features were formed under the conditions of an arid steppe climate, which contributed to the evolution of resistance to thermal stress and adaptation to regional environmental specifics. The present study enhances our understanding of the genetic architecture underlying productive and adaptive traits in local sheep breeds and underscores the need for their further investigation and conservation as a valuable reservoir of genetic diversity and biological resilience. A promising direction for future research is the structural analysis of the identified genes located in loci under selection pressure, aimed at detecting functionally significant mutations, elucidating the mechanisms of their phenotypic effects, and identifying novel productivity markers. Thus, this study may provide a foundation for the development of new marker-assisted selection strategies for the Dzhalgin Merino breed.

## Figures and Tables

**Table 1:** Weir and Cockerham weighted FST values calculated for pairwise comparisons among the Dzhalgin Merino, Australian Merino, and Rambouillet sheep groups.

	DZM	AM
**DZM**	-	-
**AM**	0.023298	-
**Ramb**	0.041518	0.050841

**Table 2:** Estimation of the significance of interactions between proteins in the constructed protein-protein networks.

Dataset	PPI Enrichment *p*-Value	
	DZM vs. AM	DZM vs. Ramb
*Ovis aries*	6.33 × 10^−3^	1.69 × 10^−10^
*Homo sapiens*	5.48 × 10^−8^	4.38 × 10^−6^

## Data Availability

The authors affirm that all data necessary for confirming the conclusions of the article are present within the article, figures, and tables.

## Supplementary Materials

The following supporting information can be downloaded at: https://www.mdpi.com/article/10.3390/ani15192871/s1, Figure S1: The “DZM vs. AM” protein–protein interaction (PPI) network of proteins encoded by candidate genes, constructed using the STRING database. Figure S2: The “DZM vs. Ramb” protein–protein interaction (PPI) network of proteins encoded by candidate genes, constructed using the STRING database. Table S1: List of candidate genes identified in loci under selection pressure in the “DZM vs. AM” comparison, confirmed by at least two independent methods. Table S2: List of candidate genes identified in loci under selection pressure in the “DZM vs. Ramb” comparison, confirmed by at least two independent methods. Table S3: GO biological process enrichment analysis for the “DZM vs. AM” candidate gene network. Table S4: GO molecular function enrichment analysis for the “DZM vs. AM” candidate gene network. Table S5: GO biological process enrichment analysis for the “DZM vs. Ramb” candidate gene network. Table S6: GO cellular component enrichment analysis for the “DZM vs. Ramb” candidate gene network.

## Abbreviations

The following abbreviations are used in this manuscript:

iHS	Integrated haplotype score
xp-EHH	Cross-population extended haplotype homozygosity
LD	Linkage disequilibrium
ROH	Runs of homozygosity
DZM	Dzhalgin Merino
AM	Australian Merino
Ramb	Rambouillet
GWAS	Genome-Wide Association Study
xp-nSL	Cross-population number of Segregating sites by Length
