# Supplementary figures and images for: Selection Signatures in the Genome of Dzhalgin Merino Sheep Breed
Source: Animals (Basel). 2025 Sep 30;15(19):2871. doi: 10.3390/ani15192871 (PMC12523943; doi:10.3390/ani15192871)

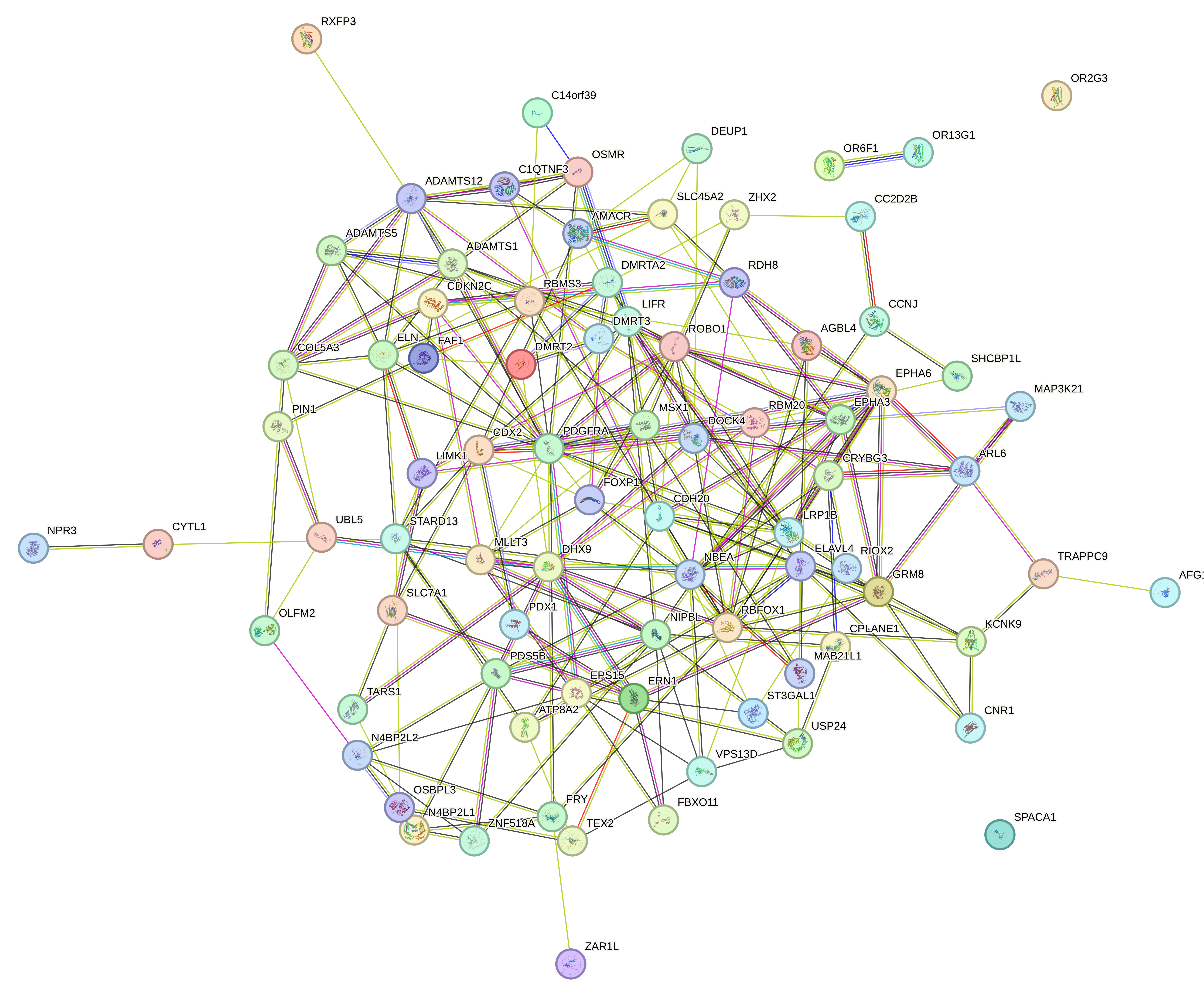

Supplement: Supplementary file 1 [file animals-15-02871-s001.zip › Figure S1.png]

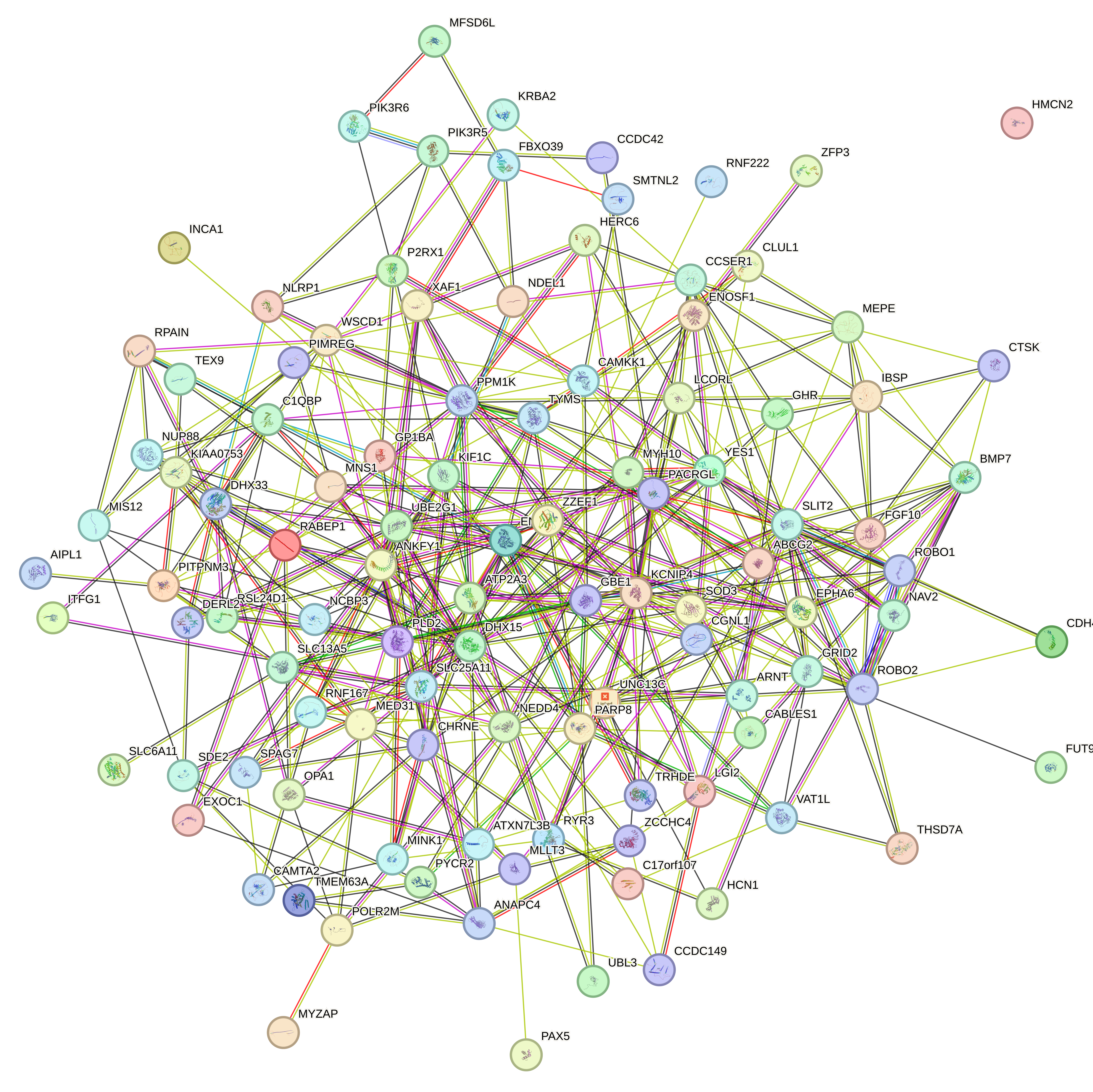

Supplement: Supplementary file 1 [file animals-15-02871-s001.zip › Figure S2 .png]
